# 4-(Dimeth­oxy­meth­yl)phenyl 2,3,4,6-tetra-*O*-acetyl-β-d-glucopyran­oside

**DOI:** 10.1107/S160053681201121X

**Published:** 2012-03-28

**Authors:** Caitlin F. Zipp, Manuel Fernandes, Helder M. Marques, Joseph P. Michael

**Affiliations:** a Molecular Sciences Institute, School of Chemistry, University of the Witwatersrand, PO Wits 2050, Johannesburg, South Africa

## Abstract

The enanti­omerically pure title compound, C_23_H_30_O_12_, crystallizes in the chiral space group *P*2_1_2_1_2_1_. The *O*-acetyl­ated-glucopyran­oside moiety adopts a chair conformation. Numerous C—H⋯O inter­actions as well as a C—H⋯π inter­action are present in the crystal structure.

## Related literature

For similar compounds, see: Bin *et al.* (2008 ▶); Ansari *et al.* (2006 ▶). For the use of sugars as water-solubilizing agents in macrocycle chemistry, see: Sol *et al.* (1997 ▶); Maillard *et al.* (1993 ▶); Oulmi *et al.* (1995 ▶). For the role of sugars in biological systems, especially proteins, see: Floyd *et al.* (2009 ▶).
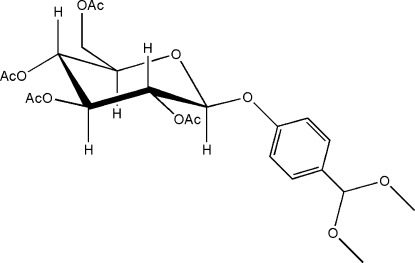


## Experimental

### 

#### Crystal data

C_23_H_30_O_12_
*M* *_r_* = 498.47
Orthorhombic, 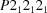

*a* = 7.9004 (2) Å
*b* = 10.9961 (3) Å
*c* = 29.2289 (8) Å
*V* = 2539.22 (12) Å^3^
*Z* = 4
Mo *K*α radiation
μ = 0.11 mm^−1^
*T* = 173 K
0.45 × 0.32 × 0.10 mm

#### Data collection

Bruker APEXII CCD diffractometer
13406 measured reflections
3493 independent reflections
2644 reflections with *I* > 2σ(*I*)
*R*_int_ = 0.053

#### Refinement

*R*[*F* ^2^ > 2σ(*F* ^2^)] = 0.042
*wR*(*F* ^2^) = 0.102
*S* = 0.97
3493 reflections
322 parameters
H-atom parameters constrained
Δρ_max_ = 0.25 e Å^−3^
Δρ_min_ = −0.20 e Å^−3^

### 

Data collection: *APEX2* (Bruker, 2005 ▶); cell refinement: *SAINT* (Bruker, 2005 ▶); data reduction: *SAINT*; program(s) used to solve structure: *SHELXS97* (Sheldrick, 2008 ▶); program(s) used to refine structure: *SHELXL97* (Sheldrick, 2008 ▶); molecular graphics: *ORTEP-3 for Windows* (Farrugia, 1997 ▶) and *SCHAKAL99* (Keller, 1999 ▶); software used to prepare material for publication: *WinGX* (Farrugia, 1999 ▶) and *PLATON* (Spek, 2009 ▶).

## Supplementary Material

Crystal structure: contains datablock(s) global, I. DOI: 10.1107/S160053681201121X/mw2057sup1.cif

Structure factors: contains datablock(s) I. DOI: 10.1107/S160053681201121X/mw2057Isup2.hkl

Additional supplementary materials:  crystallographic information; 3D view; checkCIF report

## Figures and Tables

**Table 1:** Hydrogen-bond geometry (Å, °) *Cg* is the centroid of the C7–C12 ring.

*D*—H⋯*A*	*D*—H	H⋯*A*	*D*⋯*A*	*D*—H⋯*A*
C2—H2⋯O11^i^	1.00	2.44	3.347 (3)	151
C12—H12⋯O4^ii^	0.95	2.45	3.321 (3)	152
C19—H19*A*⋯O3^iii^	0.98	2.54	3.510 (3)	171
C19—H19*C*⋯O8^iv^	0.98	2.49	3.414 (3)	157
C21—H21*C*⋯O10^iv^	0.98	2.29	3.252 (4)	168
C23—H23*C*⋯*Cg*^v^	0.98	2.71	3.615 (4)	153

Symmetry codes: (i) 
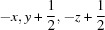
; (ii) 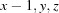; (iii) 
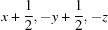
; (iv) 
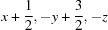
; (v) 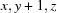.

**Fig. 1.**
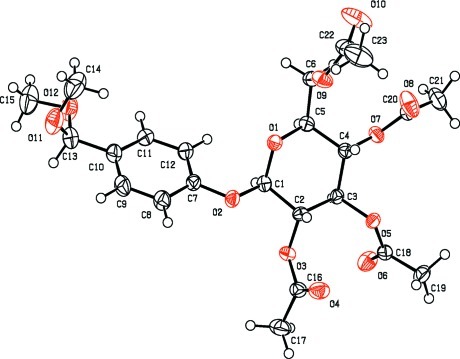
The molecular structure of (I). Displacement ellipsoids are drawn at the 50% probability level.

**Fig. 2.**
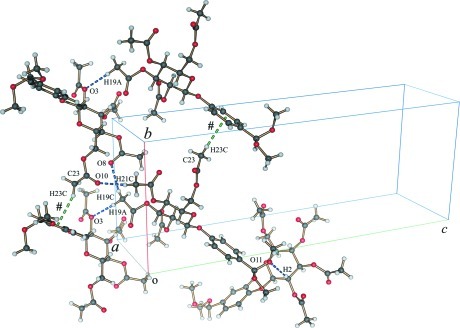
Weak hydrogen bonding in the strucuture of (I). Four of the five C—H···O interactions listed in Table 1 are shown, while the location of the C—H···π interactions is indicated with a #.

## supplementary crystallographic information

### Comment

Glycosylation is common in nature, with a large proportion of all proteins bearing sugar residues (Floyd *et al.*, 2009). The title compound was synthesized as a precursor to a biomimetic model for vitamin B12_a_ (aquacobalamin) as sugars can play a useful role in the solubilizing of large organic systems, such as glycosylated porphyrins (Sol *et al.*, 1997; Maillard *et al.*, 1993; Oulmi *et al.*, 1995).

### Experimental

The title compound was synthesized from *p*-hydroxybenzaldehyde and 2,3,4,6-tetra-*O*-acetyl-α-*D*-glucopyranosyl bromide in the presence of NaOH. The product was purified by column chromatography and recrystallized from acidified methanol to yield colourless plate-like crystals.

### Refinement

All H atoms were positioned geometrically, and allowed to ride on their parent atoms. Hydrogen bond lengths were set as follows: C—H = 0.95 Å (Aromatic C—H), 1.00 Å (Methine C—H) 0.99 Å (CH_2_) or 0.98 Å (CH_3_). Isotropic displacement parameters for these atoms were set as 1.2 times *U*_eq_ of the parent atom for CH and CH_2_, and 1.5 times *U*_eq_ of the parent atom for CH_3_.

### Crystal data

**Table:**

C_23_H_30_O_12_	*F*(000) = 1056
*M**_r_* = 498.47	*D*_x_ = 1.304 Mg m^−^^3^
Orthorhombic, *P*2_1_2_1_2_1_	Mo *K*α radiation, λ = 0.71073 Å
Hall symbol: P 2ac 2ab	Cell parameters from 3488 reflections
*a* = 7.9004 (2) Å	θ = 2.3–24.0°
*b* = 10.9961 (3) Å	µ = 0.11 mm^−^^1^
*c* = 29.2289 (8) Å	*T* = 173 K
*V* = 2539.22 (12) Å^3^	Plate, colourless
*Z* = 4	0.45 × 0.32 × 0.10 mm

### Data collection

**Table:**

Bruker APEXII CCD diffractometer	2644 reflections with *I* > 2σ(*I*)
Radiation source: fine-focus sealed tube	*R*_int_ = 0.053
Graphite monochromator	θ_max_ = 28.0°, θ_min_ = 2.0°
φ and ω scans	*h* = −10→10
13406 measured reflections	*k* = −10→14
3493 independent reflections	*l* = −38→37

### Refinement

**Table:**

Refinement on *F*^2^	Primary atom site location: structure-invariant direct methods
Least-squares matrix: full	Secondary atom site location: difference Fourier map
*R*[*F*^2^ > 2σ(*F*^2^)] = 0.042	Hydrogen site location: inferred from neighbouring sites
*wR*(*F*^2^) = 0.102	H-atom parameters constrained
*S* = 0.97	*w* = 1/[σ^2^(*F*_o_^2^) + (0.0567*P*)^2^] where *P* = (*F*_o_^2^ + 2*F*_c_^2^)/3
3493 reflections	(Δ/σ)_max_ = 0.001
322 parameters	Δρ_max_ = 0.25 e Å^−^^3^
0 restraints	Δρ_min_ = −0.20 e Å^−^^3^

### Special details

**Table:**

Geometry. All e.s.d.'s (except the e.s.d. in the dihedral angle between two l.s. planes) are estimated using the full covariance matrix. The cell e.s.d.'s are taken into account individually in the estimation of e.s.d.'s in distances, angles and torsion angles; correlations between e.s.d.'s in cell parameters are only used when they are defined by crystal symmetry. An approximate (isotropic) treatment of cell e.s.d.'s is used for estimating e.s.d.'s involving l.s. planes.
Refinement. Refinement of *F^2^* against ALL reflections. The weighted *R*- factor *wR* and goodness of fit *S* are based on *F^2^*; conventional *R*-factors *R* are based on *F*, with *F* set to zero for negative *F^2^*. The threshold expression of *F^2^* > σ(*F^2^*) is used only for calculating *R*-factors(gt) *etc*. and is not relevant to the choice of reflections for refinement. *R*-factors based on *F^2^* are statistically about twice as large as those based on *F*, and *R*-factors based on ALL data will be even larger.

### Fractional atomic coordinates and isotropic or equivalent isotropic displacement parameters (Å^2^)

**Table:**

	*x*	*y*	*z*	*U*_iso_*/*U*_eq_	
C1	0.0801 (3)	0.3057 (2)	0.12632 (8)	0.0238 (5)	
H1	0.0113	0.2457	0.1086	0.029*	
C2	0.2410 (3)	0.3384 (2)	0.10042 (8)	0.0219 (5)	
H2	0.3164	0.3905	0.1197	0.026*	
C3	0.1944 (3)	0.4037 (2)	0.05649 (8)	0.0225 (5)	
H3	0.1373	0.3461	0.0350	0.027*	
C4	0.0790 (3)	0.5105 (2)	0.06664 (8)	0.0236 (5)	
H4	0.1420	0.5753	0.0836	0.028*	
C5	−0.0749 (3)	0.4686 (2)	0.09422 (8)	0.0254 (5)	
H5	−0.1399	0.4077	0.0759	0.031*	
C6	−0.1907 (3)	0.5704 (2)	0.10783 (9)	0.0313 (6)	
H6A	−0.2358	0.6113	0.0802	0.038*	
H6B	−0.2873	0.5381	0.1257	0.038*	
C7	0.0063 (3)	0.1934 (2)	0.19310 (8)	0.0266 (5)	
C8	0.0622 (4)	0.1381 (2)	0.23282 (9)	0.0355 (7)	
H8	0.1776	0.1437	0.2417	0.043*	
C9	−0.0526 (4)	0.0745 (3)	0.25957 (9)	0.0389 (7)	
H9	−0.0149	0.0360	0.2868	0.047*	
C10	−0.2229 (4)	0.0663 (2)	0.24709 (9)	0.0349 (6)	
C11	−0.2725 (4)	0.1186 (2)	0.20664 (10)	0.0353 (6)	
H11	−0.3865	0.1100	0.1968	0.042*	
C12	−0.1597 (4)	0.1839 (2)	0.17970 (9)	0.0339 (6)	
H12	−0.1971	0.2217	0.1523	0.041*	
C13	−0.3436 (4)	−0.0023 (3)	0.27751 (10)	0.0439 (7)	
H13	−0.3008	−0.0875	0.2804	0.053*	
C14	−0.4138 (6)	0.1676 (3)	0.32407 (12)	0.0660 (11)	
H14A	−0.3371	0.2205	0.3068	0.099*	
H14B	−0.4181	0.1944	0.3560	0.099*	
H14C	−0.5274	0.1718	0.3107	0.099*	
C15	−0.6115 (6)	−0.0935 (3)	0.27581 (13)	0.0679 (11)	
H15A	−0.5587	−0.1741	0.2746	0.102*	
H15B	−0.7182	−0.0950	0.2587	0.102*	
H15C	−0.6340	−0.0716	0.3077	0.102*	
C16	0.4919 (4)	0.2178 (2)	0.09375 (8)	0.0284 (6)	
C17	0.5515 (4)	0.0911 (2)	0.08455 (10)	0.0428 (7)	
H17A	0.6752	0.0882	0.0865	0.064*	
H17B	0.5154	0.0662	0.0539	0.064*	
H17C	0.5027	0.0357	0.1073	0.064*	
C18	0.4204 (3)	0.3879 (2)	0.00160 (8)	0.0268 (5)	
C19	0.5757 (4)	0.4516 (2)	−0.01518 (10)	0.0395 (7)	
H19A	0.6324	0.4009	−0.0381	0.059*	
H19B	0.6527	0.4660	0.0106	0.059*	
H19C	0.5438	0.5296	−0.0289	0.059*	
C20	0.0206 (4)	0.6785 (2)	0.01657 (9)	0.0329 (6)	
C21	−0.0371 (5)	0.7100 (3)	−0.03023 (10)	0.0481 (8)	
H21A	−0.0692	0.7960	−0.0313	0.072*	
H21B	−0.1350	0.6597	−0.0383	0.072*	
H21C	0.0548	0.6951	−0.0520	0.072*	
C22	−0.1382 (4)	0.7733 (3)	0.13249 (11)	0.0464 (8)	
C23	−0.0184 (5)	0.8508 (3)	0.15844 (13)	0.0651 (11)	
H23A	0.0782	0.8716	0.1389	0.098*	
H23B	0.0216	0.8066	0.1855	0.098*	
H23C	−0.0759	0.9255	0.1681	0.098*	
O1	−0.0151 (2)	0.41192 (14)	0.13529 (5)	0.0264 (4)	
O2	0.1293 (2)	0.25639 (15)	0.16843 (5)	0.0283 (4)	
O3	0.3219 (2)	0.22387 (14)	0.09025 (5)	0.0257 (4)	
O4	0.5794 (3)	0.30351 (17)	0.10281 (7)	0.0401 (5)	
O5	0.3456 (2)	0.45309 (14)	0.03546 (5)	0.0249 (4)	
O6	0.3683 (3)	0.29251 (16)	−0.01228 (6)	0.0401 (5)	
O7	0.0199 (2)	0.55613 (14)	0.02296 (5)	0.0280 (4)	
O8	0.0625 (3)	0.74887 (16)	0.04572 (7)	0.0538 (6)	
O9	−0.0958 (3)	0.65541 (15)	0.13508 (6)	0.0370 (5)	
O10	−0.2594 (4)	0.8089 (2)	0.11206 (10)	0.0741 (8)	
O11	−0.3548 (3)	0.0473 (2)	0.32214 (7)	0.0566 (6)	
O12	−0.5013 (3)	−0.00697 (19)	0.25616 (7)	0.0510 (6)	

### Atomic displacement parameters (Å^2^)

**Table:**

	*U*^11^	*U*^22^	*U*^33^	*U*^12^	*U*^13^	*U*^23^
C1	0.0264 (13)	0.0235 (11)	0.0216 (12)	−0.0017 (11)	−0.0011 (11)	−0.0001 (9)
C2	0.0204 (12)	0.0216 (11)	0.0235 (12)	0.0004 (10)	0.0008 (10)	−0.0046 (9)
C3	0.0225 (12)	0.0197 (11)	0.0253 (12)	−0.0027 (10)	0.0022 (11)	−0.0003 (9)
C4	0.0265 (13)	0.0213 (11)	0.0230 (12)	0.0000 (11)	0.0013 (11)	−0.0005 (9)
C5	0.0247 (13)	0.0257 (11)	0.0259 (12)	0.0010 (10)	−0.0008 (11)	0.0006 (9)
C6	0.0295 (15)	0.0337 (13)	0.0309 (14)	0.0078 (12)	0.0020 (12)	−0.0011 (11)
C7	0.0297 (14)	0.0253 (11)	0.0248 (12)	0.0009 (11)	0.0044 (11)	0.0003 (10)
C8	0.0360 (16)	0.0430 (15)	0.0276 (14)	−0.0015 (13)	−0.0046 (13)	0.0055 (11)
C9	0.046 (2)	0.0437 (16)	0.0270 (14)	0.0015 (14)	−0.0033 (13)	0.0115 (12)
C10	0.0400 (17)	0.0347 (14)	0.0299 (14)	0.0026 (13)	0.0057 (13)	0.0056 (11)
C11	0.0287 (14)	0.0391 (14)	0.0382 (16)	0.0037 (12)	0.0046 (13)	0.0074 (12)
C12	0.0332 (16)	0.0394 (14)	0.0290 (14)	0.0050 (13)	0.0043 (12)	0.0097 (11)
C13	0.0493 (19)	0.0431 (16)	0.0393 (17)	0.0007 (15)	0.0052 (15)	0.0149 (13)
C14	0.074 (3)	0.065 (2)	0.059 (2)	−0.012 (2)	0.029 (2)	−0.0096 (17)
C15	0.076 (3)	0.062 (2)	0.066 (2)	−0.023 (2)	0.017 (2)	0.0088 (18)
C16	0.0278 (14)	0.0338 (13)	0.0237 (13)	0.0067 (12)	−0.0010 (11)	0.0011 (10)
C17	0.0434 (18)	0.0376 (14)	0.0474 (17)	0.0151 (14)	0.0015 (15)	−0.0033 (13)
C18	0.0295 (13)	0.0244 (11)	0.0265 (13)	0.0036 (11)	0.0040 (12)	0.0005 (9)
C19	0.0407 (16)	0.0323 (13)	0.0455 (16)	0.0004 (13)	0.0207 (15)	−0.0010 (12)
C20	0.0327 (15)	0.0252 (12)	0.0409 (15)	0.0023 (11)	0.0092 (13)	0.0066 (11)
C21	0.067 (2)	0.0324 (14)	0.0452 (17)	0.0067 (15)	0.0005 (17)	0.0119 (13)
C22	0.052 (2)	0.0367 (15)	0.0510 (18)	0.0163 (15)	0.0011 (17)	−0.0032 (14)
C23	0.077 (3)	0.0392 (16)	0.079 (3)	0.0126 (19)	−0.018 (2)	−0.0202 (17)
O1	0.0287 (10)	0.0289 (8)	0.0217 (8)	0.0038 (7)	0.0024 (8)	0.0015 (7)
O2	0.0272 (10)	0.0340 (9)	0.0236 (9)	−0.0010 (8)	0.0008 (8)	0.0046 (7)
O3	0.0257 (10)	0.0223 (8)	0.0291 (9)	0.0039 (7)	−0.0011 (8)	−0.0021 (7)
O4	0.0261 (10)	0.0378 (10)	0.0564 (13)	0.0012 (9)	−0.0027 (10)	−0.0023 (9)
O5	0.0251 (9)	0.0213 (7)	0.0283 (9)	−0.0018 (7)	0.0070 (8)	−0.0022 (7)
O6	0.0433 (12)	0.0332 (10)	0.0439 (11)	−0.0046 (9)	0.0128 (10)	−0.0138 (8)
O7	0.0336 (10)	0.0221 (8)	0.0283 (9)	0.0029 (7)	−0.0002 (8)	0.0025 (7)
O8	0.0837 (18)	0.0234 (9)	0.0542 (13)	−0.0053 (11)	−0.0061 (13)	0.0034 (9)
O9	0.0466 (12)	0.0302 (9)	0.0343 (10)	0.0134 (9)	−0.0038 (10)	−0.0072 (7)
O10	0.0699 (18)	0.0433 (12)	0.109 (2)	0.0250 (13)	−0.0303 (16)	−0.0047 (13)
O11	0.0628 (16)	0.0706 (15)	0.0365 (12)	−0.0076 (14)	0.0116 (11)	0.0106 (11)
O12	0.0476 (13)	0.0543 (13)	0.0511 (13)	−0.0148 (12)	0.0048 (11)	0.0135 (10)

### Geometric parameters (Å, º)

**Table:**

C1—O2	1.400 (3)	C13—H13	1.0000
C1—O1	1.414 (3)	C14—O11	1.404 (4)
C1—C2	1.522 (3)	C14—H14A	0.9800
C1—H1	1.0000	C14—H14B	0.9800
C2—O3	1.444 (3)	C14—H14C	0.9800
C2—C3	1.516 (3)	C15—O12	1.412 (4)
C2—H2	1.0000	C15—H15A	0.9800
C3—O5	1.449 (3)	C15—H15B	0.9800
C3—C4	1.516 (3)	C15—H15C	0.9800
C3—H3	1.0000	C16—O4	1.198 (3)
C4—O7	1.449 (3)	C16—O3	1.348 (3)
C4—C5	1.530 (3)	C16—C17	1.495 (3)
C4—H4	1.0000	C17—H17A	0.9800
C5—O1	1.433 (3)	C17—H17B	0.9800
C5—C6	1.500 (3)	C17—H17C	0.9800
C5—H5	1.0000	C18—O6	1.198 (3)
C6—O9	1.439 (3)	C18—O5	1.357 (3)
C6—H6A	0.9900	C18—C19	1.495 (4)
C6—H6B	0.9900	C19—H19A	0.9800
C7—C12	1.373 (4)	C19—H19B	0.9800
C7—C8	1.383 (4)	C19—H19C	0.9800
C7—O2	1.394 (3)	C20—O8	1.198 (3)
C8—C9	1.387 (4)	C20—O7	1.359 (3)
C8—H8	0.9500	C20—C21	1.483 (4)
C9—C10	1.397 (4)	C21—H21A	0.9800
C9—H9	0.9500	C21—H21B	0.9800
C10—C11	1.372 (4)	C21—H21C	0.9800
C10—C13	1.506 (4)	C22—O10	1.195 (4)
C11—C12	1.390 (4)	C22—O9	1.341 (3)
C11—H11	0.9500	C22—C23	1.482 (5)
C12—H12	0.9500	C23—H23A	0.9800
C13—O12	1.395 (4)	C23—H23B	0.9800
C13—O11	1.417 (4)	C23—H23C	0.9800
			
O2—C1—O1	107.73 (17)	O11—C13—H13	107.8
O2—C1—C2	107.29 (19)	C10—C13—H13	107.8
O1—C1—C2	109.94 (18)	O11—C14—H14A	109.5
O2—C1—H1	110.6	O11—C14—H14B	109.5
O1—C1—H1	110.6	H14A—C14—H14B	109.5
C2—C1—H1	110.6	O11—C14—H14C	109.5
O3—C2—C3	110.26 (18)	H14A—C14—H14C	109.5
O3—C2—C1	105.40 (17)	H14B—C14—H14C	109.5
C3—C2—C1	109.29 (19)	O12—C15—H15A	109.5
O3—C2—H2	110.6	O12—C15—H15B	109.5
C3—C2—H2	110.6	H15A—C15—H15B	109.5
C1—C2—H2	110.6	O12—C15—H15C	109.5
O5—C3—C4	106.76 (17)	H15A—C15—H15C	109.5
O5—C3—C2	109.66 (19)	H15B—C15—H15C	109.5
C4—C3—C2	110.31 (18)	O4—C16—O3	123.5 (2)
O5—C3—H3	110.0	O4—C16—C17	126.2 (3)
C4—C3—H3	110.0	O3—C16—C17	110.2 (2)
C2—C3—H3	110.0	C16—C17—H17A	109.5
O7—C4—C3	106.83 (17)	C16—C17—H17B	109.5
O7—C4—C5	108.2 (2)	H17A—C17—H17B	109.5
C3—C4—C5	110.31 (18)	C16—C17—H17C	109.5
O7—C4—H4	110.5	H17A—C17—H17C	109.5
C3—C4—H4	110.5	H17B—C17—H17C	109.5
C5—C4—H4	110.5	O6—C18—O5	124.0 (2)
O1—C5—C6	107.67 (18)	O6—C18—C19	125.5 (2)
O1—C5—C4	108.10 (19)	O5—C18—C19	110.4 (2)
C6—C5—C4	113.56 (19)	C18—C19—H19A	109.5
O1—C5—H5	109.1	C18—C19—H19B	109.5
C6—C5—H5	109.1	H19A—C19—H19B	109.5
C4—C5—H5	109.1	C18—C19—H19C	109.5
O9—C6—C5	108.3 (2)	H19A—C19—H19C	109.5
O9—C6—H6A	110.0	H19B—C19—H19C	109.5
C5—C6—H6A	110.0	O8—C20—O7	122.9 (2)
O9—C6—H6B	110.0	O8—C20—C21	126.2 (2)
C5—C6—H6B	110.0	O7—C20—C21	110.9 (2)
H6A—C6—H6B	108.4	C20—C21—H21A	109.5
C12—C7—C8	120.7 (2)	C20—C21—H21B	109.5
C12—C7—O2	123.8 (2)	H21A—C21—H21B	109.5
C8—C7—O2	115.5 (2)	C20—C21—H21C	109.5
C7—C8—C9	119.1 (3)	H21A—C21—H21C	109.5
C7—C8—H8	120.5	H21B—C21—H21C	109.5
C9—C8—H8	120.5	O10—C22—O9	123.0 (3)
C8—C9—C10	121.0 (2)	O10—C22—C23	125.4 (3)
C8—C9—H9	119.5	O9—C22—C23	111.6 (3)
C10—C9—H9	119.5	C22—C23—H23A	109.5
C11—C10—C9	118.2 (3)	C22—C23—H23B	109.5
C11—C10—C13	122.5 (3)	H23A—C23—H23B	109.5
C9—C10—C13	119.2 (2)	C22—C23—H23C	109.5
C10—C11—C12	121.5 (3)	H23A—C23—H23C	109.5
C10—C11—H11	119.3	H23B—C23—H23C	109.5
C12—C11—H11	119.3	C1—O1—C5	112.28 (16)
C7—C12—C11	119.4 (2)	C7—O2—C1	117.00 (19)
C7—C12—H12	120.3	C16—O3—C2	117.93 (19)
C11—C12—H12	120.3	C18—O5—C3	118.07 (18)
O12—C13—O11	111.7 (3)	C20—O7—C4	117.55 (19)
O12—C13—C10	108.7 (2)	C22—O9—C6	117.8 (2)
O11—C13—C10	113.0 (2)	C14—O11—C13	114.9 (2)
O12—C13—H13	107.8	C13—O12—C15	113.2 (3)
			
O2—C1—C2—O3	67.0 (2)	C11—C10—C13—O11	−121.4 (3)
O1—C1—C2—O3	−176.06 (17)	C9—C10—C13—O11	60.5 (4)
O2—C1—C2—C3	−174.46 (17)	O2—C1—O1—C5	−178.82 (18)
O1—C1—C2—C3	−57.6 (2)	C2—C1—O1—C5	64.6 (2)
O3—C2—C3—O5	−74.1 (2)	C6—C5—O1—C1	173.20 (19)
C1—C2—C3—O5	170.47 (17)	C4—C5—O1—C1	−63.7 (2)
O3—C2—C3—C4	168.59 (18)	C12—C7—O2—C1	−4.1 (3)
C1—C2—C3—C4	53.2 (2)	C8—C7—O2—C1	175.4 (2)
O5—C3—C4—O7	69.5 (2)	O1—C1—O2—C7	76.8 (2)
C2—C3—C4—O7	−171.43 (18)	C2—C1—O2—C7	−164.91 (18)
O5—C3—C4—C5	−173.12 (18)	O4—C16—O3—C2	−3.5 (4)
C2—C3—C4—C5	−54.0 (3)	C17—C16—O3—C2	176.82 (19)
O7—C4—C5—O1	174.03 (16)	C3—C2—O3—C16	101.6 (2)
C3—C4—C5—O1	57.5 (2)	C1—C2—O3—C16	−140.5 (2)
O7—C4—C5—C6	−66.6 (2)	O6—C18—O5—C3	0.7 (3)
C3—C4—C5—C6	176.9 (2)	C19—C18—O5—C3	−178.8 (2)
O1—C5—C6—O9	60.3 (2)	C4—C3—O5—C18	−143.3 (2)
C4—C5—C6—O9	−59.4 (3)	C2—C3—O5—C18	97.2 (2)
C12—C7—C8—C9	−1.2 (4)	O8—C20—O7—C4	−2.4 (4)
O2—C7—C8—C9	179.4 (2)	C21—C20—O7—C4	177.8 (2)
C7—C8—C9—C10	−0.4 (4)	C3—C4—O7—C20	−135.4 (2)
C8—C9—C10—C11	2.7 (4)	C5—C4—O7—C20	105.8 (2)
C8—C9—C10—C13	−179.2 (3)	O10—C22—O9—C6	6.9 (5)
C9—C10—C11—C12	−3.5 (4)	C23—C22—O9—C6	−173.9 (3)
C13—C10—C11—C12	178.5 (3)	C5—C6—O9—C22	148.1 (2)
C8—C7—C12—C11	0.4 (4)	O12—C13—O11—C14	−61.7 (3)
O2—C7—C12—C11	179.8 (2)	C10—C13—O11—C14	61.2 (4)
C10—C11—C12—C7	2.0 (4)	O11—C13—O12—C15	−69.5 (3)
C11—C10—C13—O12	3.2 (4)	C10—C13—O12—C15	165.2 (3)
C9—C10—C13—O12	−174.9 (3)		

### Hydrogen-bond geometry (Å, º)

Cg is the centroid of the C7–C12 ring.

**Table:**

*D*—H···*A*	*D*—H	H···*A*	*D*···*A*	*D*—H···*A*
C2—H2···O11^i^	1.00	2.44	3.347 (3)	151
C12—H12···O4^ii^	0.95	2.45	3.321 (3)	152
C19—H19*A*···O3^iii^	0.98	2.54	3.510 (3)	171
C19—H19*C*···O8^iv^	0.98	2.49	3.414 (3)	157
C21—H21*C*···O10^iv^	0.98	2.29	3.252 (4)	168
C23—H23*C*···*Cg*^v^	0.98	2.71	3.615 (4)	153

Symmetry codes: (i) −*x*, *y*+1/2, −*z*+1/2; (ii) *x*−1, *y*, *z*; (iii) *x*+1/2, −*y*+1/2, −*z*; (iv) *x*+1/2, −*y*+3/2, −*z*; (v) *x*, *y*+1, *z*.
